# Immunomodulatory strategies prevent the development of autoimmune emphysema

**DOI:** 10.1186/1465-9921-11-179

**Published:** 2010-12-16

**Authors:** Masayuki Hanaoka, Mark R Nicolls, Andrew P Fontenot, Donatas Kraskauskas, Douglas G Mack, Adelheid Kratzer, Jonas Salys, Vita Kraskauskiene, Nana Burns, Norbert F Voelkel, Laimute Taraseviciene-Stewart

**Affiliations:** 1 Department of Medicine, University of Colorado Denver, Aurora, CO, 80045, USA; 2 VA Palo Alto Health Care System, Stanford University, Palo Alto, CA, 94304, USA; 3 Pulmonary and Critical Care Medicine Division and Virginia Johnson Center for Emphysema Research, Virginia Commonwealth University, Richmond, VA, 23298, USA

## Abstract

**Background:**

The presence of anti-endothelial cell antibodies and pathogenic T cells may reflect an autoimmune component in the pathogenesis of emphysema. Whether immune modulatory strategies can protect against the development of emphysema is not known.

**Methods:**

Sprague Dawley rats were immunized with human umbilical vein endothelial cells (HUVEC) to induce autoimmune emphysema and treated with intrathymic HUVEC-injection and pristane. Measurements of alveolar airspace enlargement, cytokine levels, immuno histochemical, western blot analysis, and T cell repertoire of the lung tissue were performed.

**Results:**

The immunomodulatory strategies protected lungs against cell death as demonstrated by reduced numbers of TUNEL and active caspase-3 positive cells and reduced levels of active caspase-3, when compared with lungs from HUVEC-immunized rats. Immunomodulatory strategies also suppressed anti-endothelial antibody production and preserved CNTF, IL-1alpha and VEGF levels. The immune deviation effects of the intrathymic HUVEC-injection were associated with an expansion of CD4+CD25+Foxp3+ regulatory T cells. Pristane treatment decreased the proportion of T cells expressing receptor beta-chain, Vβ16.1 in the lung tissue.

**Conclusions:**

Our data demonstrate that interventions classically employed to induce central T cell tolerance (thymic inoculation of antigen) or to activate innate immune responses (pristane treatment) can prevent the development of autoimmune emphysema.

## Background

It is now increasingly recognized that chronic obstructive pulmonary disease (COPD)/emphysema presents clinically as a syndrome with pulmonary and extra-pulmonary manifestations [1,2]. Chronic inflammatory mechanisms are being discussed as important factors which either trigger or maintain this chronic disorder [3-5]. Both lymphocytic infiltration of the bronchial mucosa and lung parenchyma [6,7] as well as lymph follicles in close proximity to bronchioles have been described [8]. It has been hypothesized that there may be an autoimmune component to the chronic progressive lung tissue destruction which can persist after smoking cessation [9-12]. Recently Lee and co-workers demonstrated anti-elastin autoantibodies in patients with tobacco smoking-induced emphysema [13]. Autoimmune diseases result from the propagation of self-reactive T and B lymphocytes that recognize self-antigens and mediate tissue destruction.

Our group has described a rat autoimmune emphysema model which is characterized by anti-endothelial cell antibodies (AECA) and pathologic T lymphocytes, since both plasma as well as splenocytes have been shown to trigger emphysema after passive transfer into healthy naïve rodents [14], suggesting that the pre-existing T cell repertoire in the lung is not sufficient to respond to xenogeneic antigens. We reasoned that this autoimmune model of emphysema, which is based on immunization of rats with xenogeneic human umbilical vein endothelial cells (HUVEC), can be utilized to demonstrate the phenomenon of immune tolerance, because autoimmunity is due to the breakdown of central and/or peripheral tolerance. If so, then demonstration of protection against emphysematous lung destruction by immune modulation would provide distinct support for the role of an autoimmune contribution to lung destruction in this rodent model [14]. This concept has been repeatedly demonstrated in the NOD (non-obese diabetic) model of autoimmune diabetes in which multiple interventions in young mice skew the immune response and prevent disease [15,16].

In the present study, we utilize interventions classically employed in experimental autoimmune models to induce central T cell tolerance by thymic inoculation of antigen [17] and to activate innate immune responses with pristane [18]. The acquisition of T cell tolerance to self occurs primarily within the thymus [19,20] and can be achieved through 3 not mutually exclusive mechanisms: clonal deletion, clonal anergy and specific suppression. Intrathymic tolerance induction by inoculation of donor bone marrow cells, spleen cells and other type of cells has been successfully used to prolong the survival of cardiac, skin and islet allografts in adult rodents [20-23]. Here we expand previous studies by utilizing an intrathymic inoculation approach in our animal model of autoimmune emphysema. The hydrocarbon oil adjuvant pristane has been shown to induce a persistent decrease in phytohemagglutinin responsiveness, consistent with a block of polyclonal T cell proliferation [18]. By demonstrating that early immunomodulation prevents experimental emphysema in pathogen-free animals, the concept that autoimmune injury may contribute to clinical emphysema is further bolstered.

## Methods

### Experimental protocols

The experimental protocol was approved by the Animal Care and Use Committee of the University of Colorado Denver. In our study we used 6 week old male Sprague-Dawley rats (body weight 200 g). Animals were divided into four groups (n = 6 per group): (1) control group (IP injection of phosphate buffered saline (PBS)), (2) HUVEC (H group) alone (10 × 10^6 ^cells, single IP injection[14]), (3) intrathymic injection of HUVEC (H-T-H group) (200,000 cells) 2 weeks prior to immunization with HUVEC; (4) pristane (H-P group) (200 μl, single IP injection) 3 days prior to immunization with HUVEC. Pristane (2,6,10,14-tetramethyl-pentadecane) was purchased from Sigma-Aldrich Co. (St. Louis, MO). While in our previous study we performed immunization with adjuvant Titermax Gold, later we found out that HUVEC itself are strong immunogens and no adjuvant is needed for antibody production. In the present study we omitted this step of adjuvant administration.

Primary cultures of HUVEC were isolated in our laboratory following procedures described by Bruneel *et al *[24].

### Intrathymic injections

Intrathymic injections were performed under anesthesia induced with a combination of ketamine(100 mg/kg)/xylazine (15 mg/kg). The thymus gland was exposed through a small incision above the sternum and 200,000 HUVEC cells in 100 μl of PBS were injected into thymic lobes (50 μl/per lobe). The control group received only PBS injections. The incision was closed with a 4-0 nylon suture. The injections were performed into 6 week old rats (body weight 200 g) 2 weeks prior to immunization with HUVEC. Indeed, these are very young rats and prior literature [25-27] supports intrathymic injection at 6 weeks.

### Tissue processing

All the animals were sacrificed 3 weeks after the IP HUVEC treatment or PBS injection [14]. Lung tissue was obtained by opening the chest wall. Lungs were ligated. The right lung was inflated with 0.5% low melting agarose at a constant pressure of 25 cmH_2_O and fixed in 10% formalin for 48 hr. Lung sections were embedded in paraffin using standard techniques. The upper and lower lobes of the left lung were snap-frozen for protein extraction.

### Lung morphology

The 5 μm thick sections were stained with hematoxylin and eosin. Ten images per slide were acquired at x200 magnification with an AxioCam Color Camera using an Axioskop 2 Microscope (Carl Zeiss MicroImaging Inc., Thornwood, NY). The degree of emphysema was quantified by measuring alveolar airspaces (in pixels per square micrometer [14]) and mean linear intercept (MLI; [28]). The CD4+ cell accumulation areas were measured in pixels per square micrometers. A macro was created to automate the segmentation, processing, and quantification steps. For each animal, 10 fields at a magnification of x100 were captured in a blinded fashion using Carl Zeiss KS300 Imaging System software.

### Cell death assessment and western blot (WB) analysis

Terminal deoxynucleotidyl transferase-mediated dUTP nick end-labeling (TUNEL) was performed with TACS 2 TdT DAB kit (Trevigen, Gaithersburg, MD) following manufacturer's protocol. The ratio of TUNEL-positive cells to total cells (apoptotic index) was measured assessing more than 3,000 parenchymal cells of each lung sample in each group [29].

Active caspase-3 was assessed using a rabbit polyclonal antibody to cleaved caspase-3 (Cell Signaling Technology Inc., Beverly, MA). Immunohistochemical staining was performed using VECTASTAIN ABC kit (Vector Laboratories Inc., Burlingame, CA).

WB analysis was performed as previously described [14]. Anti-CD19 antibodies were from Santa Cruz Biotechnology Inc., Santa Cruz, CA, and anti-CD68 from Serotec Ltd, Oxford, UK.

### Cytokine assay

Cytokine levels in whole lung tissue homogenates were determined using Quantibody Rat-1 Array (RayBiotech, Inc, Norcross, GA) that allows measurement of the following cytokines: CINC-2 (CXCL3/GROgamma/DCIP-1), CINC-3 (CXCL3/GRObeta/MIP-2), Fractalkine (CX3CL1), CNTF, GM-CSF, IL-1α, IL-4, LIX (CXCL5), β-NGF and VEGF.

### Lung and spleen T cell isolation

Cells were isolated as described by Finotto et al., [30]. Negative selection of CD4+ T cells was performed using Rat CD4+ T Cell Enrichment Column from R&D Systems, Inc. (Minneapolis, MN). The aliquot of the sorted cell preparation (100 μl) was cytospinned, stained with Wright stain and counted. The CD4+ T cells isolated from the lungs were identified by flow cytometry. We also did the CD3 staining to confirm that the CD4+ cells were T cells and not, for example, strange macrophages.

### Lung CD4+ T cell measurements

Lung sections were stained with an anti-CD4 antibody and CD4+ cells were counted in 5 different lung fields. The CD4+ cell accumulation areas were measured in pixels per square micrometers. A macro was created to automate the segmentation, processing, and quantification steps using a Carl Zeiss KS300 Imaging System software. A CD4+ T cell count was also performed on cells isolated from the lungs. A CD4+ T cell count was also performed on cells isolated from the lungs by flow cytometry. Both methods gave comparable results.

### Flow cytometry

Isolated CD4+ T cells were washed in PBS supplemented with 1% bovine serum albumin (BSA) (FACS buffer) and surface stained with mAbs directed against CD3 (FITC, Invitrogen) and CD4 (APC, Invitrogen or BD) along with one of the following mAbs to TCR V beta (8.5, 10, or 16.1 (AbD Serotec) or CD25 (PE, Biolegend) for 30 min at 4°C as described previously [31]. Cells were washed in FACS buffer and stained with secondary antibodies to each TCR V beta (R-PE, Invitrogen). For intranuclear staining with anti-Foxp3 (PE; eBiosciences), surface stained cells were washed twice, fixed and permeabilized prior to staining with anti-Foxp3. Cells were washed and resuspended in 1% formaldehyde. The number of events collected ranged between 1 and 3 million. Samples were acquired on a FACSCalibur equipped with 488 nm argon and 633 nm red-diode laser for four color detection. Analysis was performed using FlowJo (Treestar) software. Lymphocytes were gated using forward scatter versus side scatter.

### Statistical analysis

All data are expressed as mean ± SEM. Statistical analysis was performed with the Statview software package (SAS Institute Inc., Cary, NC). The comparison between two groups was performed by employing the Student's unpaired t test for independent samples. The values obtained in the different groups of rats were compared using one-way ANOVA. Statistical difference was accepted at p < 0.05.

## Results

### Immunomodulation strategies protect against airspace enlargement

**Figure 1:**
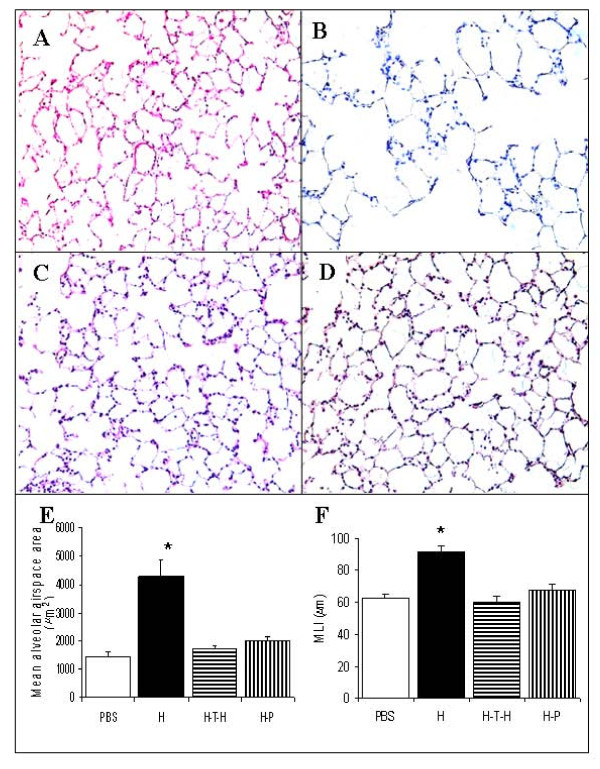
**Morphology of rat lungs and alveolar airspace measurements**. Hematoxylin and eosin (H&E) staining of rat lungs (n = 6 in each group): (**A**) PBS-treated control (PBS); (**B**) HUVEC-immunized (H); (**C**) Intrathymically HUVEC-injected and HUVEC-immunized (H-T-H); (**D**) Pristane-treated 3 days prior to HUVEC-immunization (H-P). (**E**) Quantitation of mean alveolar airspace area (micrometer^2^); (**F**) Mean linear intercept (MLI). Magnification x200.*p < 0.05 compared to control and each of tolerized rat groups.

As previously reported [14], when compared to lungs from PBS-injected control animals (Figure 1A), histological examination of lung tissue samples demonstrated diffusely enlarged airspaces in HUVEC-immunized rats (Figure 1B). In contrast, there was no alveolar space enlargement in the lungs from animals treated with either intrathymic HUVEC-injection (Figure 1C) or pristane (Figure 1D) in association with intraperitoneal HUVEC injection. The quantitative data of alveolar airspace surface area and mean linear intercept (MLI) from all treatment groups are shown in Figure 1E and Figure 1F. The sections evaluated were selected in a random fashion.

### Immunomodulation strategies protect against lung cell apoptosis

**Figure 2:**
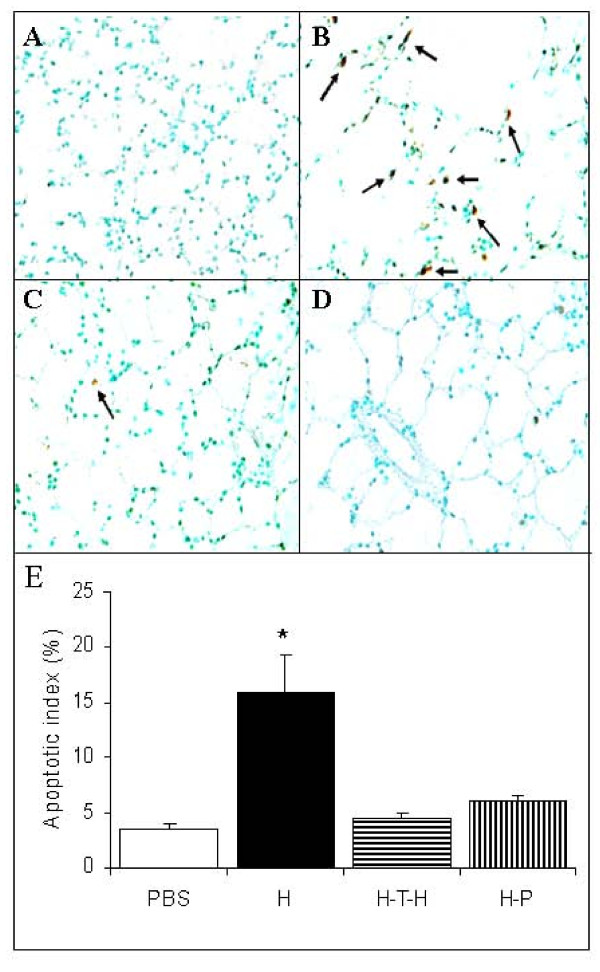
**Apoptosis in rat lungs and apoptotic index**. Cell death in rat lungs (n = 6) assessed by TUNEL staining. (**A**) PBS-treated control; (**B**) HUVEC-immunized; (**C**) Intrathymically HUVEC-injected and HUVEC-immunized; (**D**) Pristane-treated 3 days prior to HUVEC-immunization. (**E**) The apoptotic index (%) was calculated as the ratio of TUNEL-positive cells to total lung cells. Magnification x400. *p < 0.05 compared to control and each of tolerized rat groups.

Both of the immunomodulatory treatments protected lungs against cell death as shown by the reduced numbers of TUNEL positive cells. Figure 2A shows PBS-treated control rat lung. When compared to lungs from HUVEC-immunized rats (Figure 2B), intrathymic injection of HUVEC followed by HUVEC immunization (Figure 2C) and pristane treatment (Figure 2D) significantly reduced TUNEL-positive cells in the rat lungs. The quantitative analysis of TUNEL positive cells is shown as an apoptotic index (Figure 2E).

### Immunomodulation strategies reduce lung inflammation and prevent caspase-3 activation

**Figure 3:**
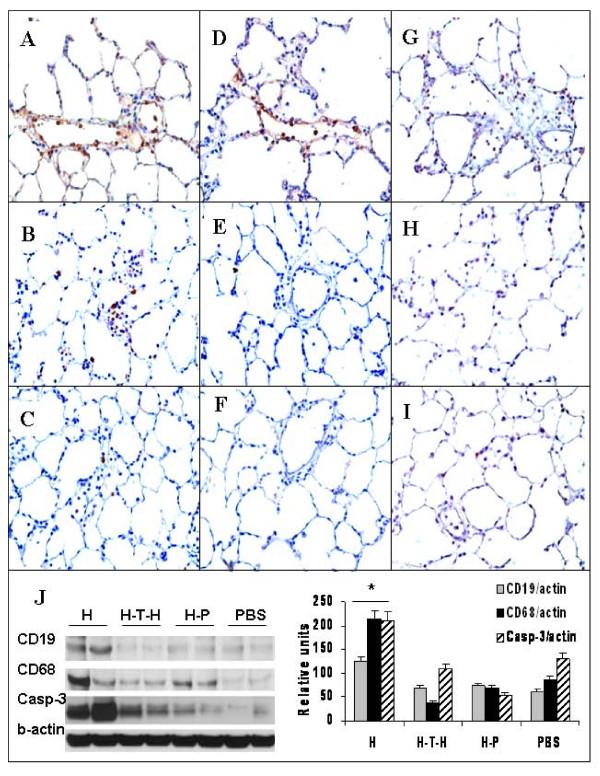
**Immunostaining for B cells (CD19), macrophages (CD68) and active caspase-3 and western blot (WB) analysis**. CD19 (**A-C**); CD68 (**D-F**) and active caspase-3 (**G-I**) of HUVEC-immunized (**A, D, G**); intrathymically HUVEC-injected and HUVEC-immunized (**B, E, F**); and pristane-treated 3 days prior to HUVEC-immunization (**C, F, I**) rat lung sections. Magnification x400. (**J**) Quantitative WB analysis of rat lung tissue homogenates for CD19, CD68 and active caspase-3. *p < 0.01 compared to control or immune tolerized rats.

Earlier we have reported [14] that during the first week of the immunization there was no difference in the numbers of CD19 or CD68 cells in immunized or control rat lungs. However, the accumulation of B cells (CD19+; Figure 3A) was observed in rat lungs at 1.5-3 weeks after HUVEC-injection. The number of these cells in the lung tissue was attenuated by intrathymic HUVEC-injection (Figure 3B) and pristane treatment (Figure 3C). The accumulation of macrophages as shown by immunohistochemistry for CD68 in HUVEC-immunized rat lungs (Figure 3D) was reduced by intrathymic HUVEC-injection (Figure 3E) and pristane-treatment (Figure 3F).

Because TUNEL staining detects both apoptotic and necrotic cells, to identify apoptotic events in the lung tissue, we performed immunohistochemistry (IHC) for active caspase-3 (Figure 3G - HUVEC-immunized; Figure 3H - intrathymic HUVEC-injection and Figure 3I pristane-treated rat lungs). The number of active caspase-3 positive cells was markedly reduced by both of the tolerizing strategies.

We confirmed the IHC findings by performing quantitative Western blot (WB) analysis of whole lung tissue extracts. The levels of activated caspase-3, CD68 and CD19 were significantly higher in the HUVEC-immunized rats compared to the PBS-treated controls (Figure 3J). The induction of central tolerance with intrathymic HUVEC-injection and the immunomodulatory strategy using pristane attenuated the expression of caspase-3 as well as the expression of CD68 and CD19.

### Immunomodulation and T cells

**Figure 4:**
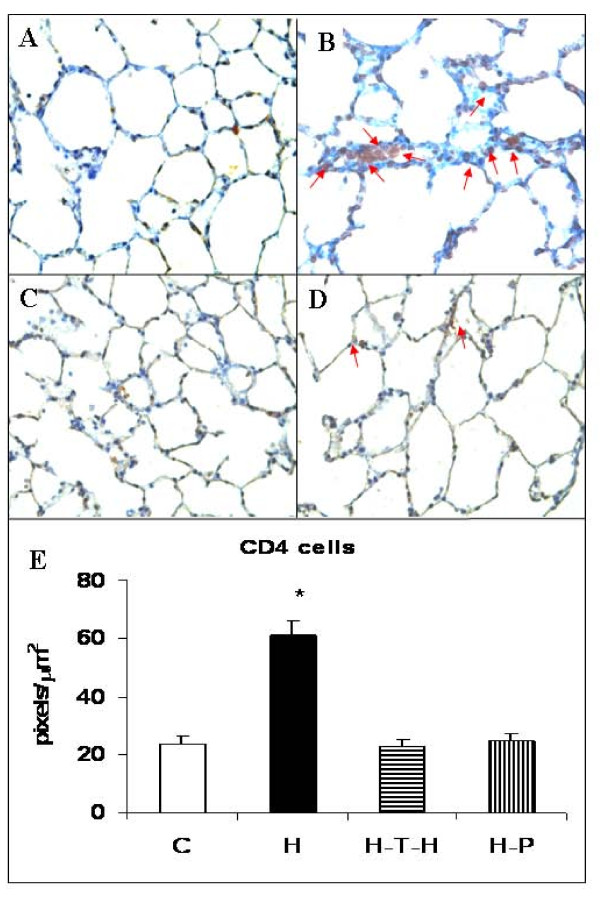
**Immunostaining for CD4+ T cells**. Immunohistochemistry for CD4+ T cells in PBS-treated (**A**); HUVEC-immunized (**B**); intrathymically HUVEC-injected and HUVEC-immunized (**C**); and pristane-treated 3 days prior to HUVEC-immunization (**D**) rat lung sections. Magnification x400. (**E**) The CD4+ cell accumulation areas were measured in pixels per square micrometers. A macro was created to automate the segmentation, processing, and quantification steps. For each animal (n = 4), 10 fields at a magnification of x100 were captured in a blinded fashion using Carl Zeiss KS300 Imaging System software. *p ≤ 0.002 when compared to immune tolerized rats.

Usually, in normal rat lung, very few CD4+ T cells can be found (Figure 4A). Conversely, in HUVEC-injected rat lungs there is a significant accumulation of CD4+ T cells (Figure 4B). Tolerizing strategies such as thymic injection of antigen (Figure 4C) and pristane treatment (Figure 4D) reduced the CD4+ cell numbers to the normal level. We have quantified the accumulation of CD4+ cells on IHC slides using the Carl Zeiss KS300 Imaging System software. As shown in Figure 4E, there was almost a 3-fold increase in the CD4+ T cell numbers in the HUVEC-immunized rat lungs when compared to untreated controls or rats treated with thymic injection of antigen or with pristane lungs. CD4+ cells from 4 rats per group were counted.

### Pristane treatment suppresses specific T cell receptor (TCR) expression

Pristane treatment decreased numbers of CD4+ T cells in the lung tissue. Here we have tested whether pristane treatment affected T cell receptor expression in the lung by testing the expression of TCR Vβ16.1, TCR Vβ8.5 and TCR Vβ10 in the treated and untreated HUVEC-immunized rat lungs. There was a higher expression of TCR Vβ8.5 in lungs and spleens from pristane-treated and HUVEC-immunized rats when compared to normal controls. The expression of TCR Vβ10 was similar in all 3 groups. While there was no significant difference in TCR Vβ expression on CD4+ T cells obtained from lung and spleen of normal controls and HUVEC-immunized animals (n = 4 rats per group), there was a significantly decreased expression of Vβ16.1 in both lungs (p ≤ 0.008) and spleens (p ≤ 0.003) of pristane treated rats compared to rats receiving only HUVEC (Table 1). Similar findings were seen with the intrathymic injection of HUVEC prior to IP immunization in lungs and spleen (9.03 ± 0.94% and 8.83 ± 0.47% respectively) when compared to HUVEC-immunized rats (11.5 ± 0.88% and 12.1 ± 0.4%; p ≤ 0.03 and p ≤ 0.007). Since HUVEC-immunized rats have 3-times more of such CD4+ T cells in the lungs, these findings might suggest that pristane and intrathymic injection of HUVEC targeted a specific CD4+ T cell subset and raise the possibility that CD4+ T cells expressing TCR Vβ16.1 may represent the pathogenic T cell population.

**Table 1:** TCR Vβ expression on CD4+ T cells (%) from lung and spleen of HUVEC and HUVEC-pristane treated rats (n = 4).

	Vβ16.1		Vβ10		Vβ8.5	
**Experimental Groups (n = 4)**	**lung**	**spleen**	**lung**	**spleen**	**lung**	**spleen**

Control	12.1 ± 0.34	12.7 ± 0.34	9.7 ± 0.29	9.2 ± 0.59	4.2 ± 0.414	4.4 ± 0.59

HUVEC*	11.5 ± 0.88	12.1 ± 0.4	8.22 ± 0.34	8.08 ± 0.61	7.1 ± 1.27	8.4 ± 2.41

HUVEC+Pristane*	7.67 ± 0.47	9.05 ± 0.59	7.1 ± 0.77	5.62 ± 1.68	7.79 ± 1.32	5.7 ± 1.82

	*p ≤ 0.008	*p ≤ 0.003	*p ≥ 0.05	*p ≥ 0.05	*p ≥ 0.05	*p ≥ 0.05

### Intrathymic injection of antigen prior to immunization induces expansion of regulatory T (Treg) cells

Recent findings indicate that targeted deletion of Treg cells causes spontaneous autoimmune disease in mice, whereas augmentation of Treg-cell function can prevent the development of - or alleviate - variants of experimental autoimmune encephalomyelitis [32]. As shown in Table 2 levels of Foxp3+/CD25+/CD4+ Treg cells in the normal control rat lungs and spleen are extremely low. However, intrathymic injection of antigen prior to IP immunization resulted in a significantly increased percentage of Foxp3-expressing CD4+CD25+ Treg cells when compared with HUVEC-immunized rats, suggesting that the induction of central tolerance results in an expansion of naturally-occurring Treg cells (n = 4 rats per group).

**Table 2:** The percentage of Foxp3-expressing CD25+/CD4+ T regulatory cells in rat lungs and spleen. CD4+/CD25+/Foxp3+

Experimental Groups	lung	spleen
Control	0.69 ± 0.12	0.69 ± 0.03

HUVEC	38.00 ± 1.60	40.60 ± 0.52

Intrathymic-Injection-HUVEC	56.00 ± 0.38	52.7 ± 1.23

	p ≤ 0.001	p ≤ 0.001

### Pristane treatment ameliorated the increase in lung tissue cytokine levels

**Figure 5:**
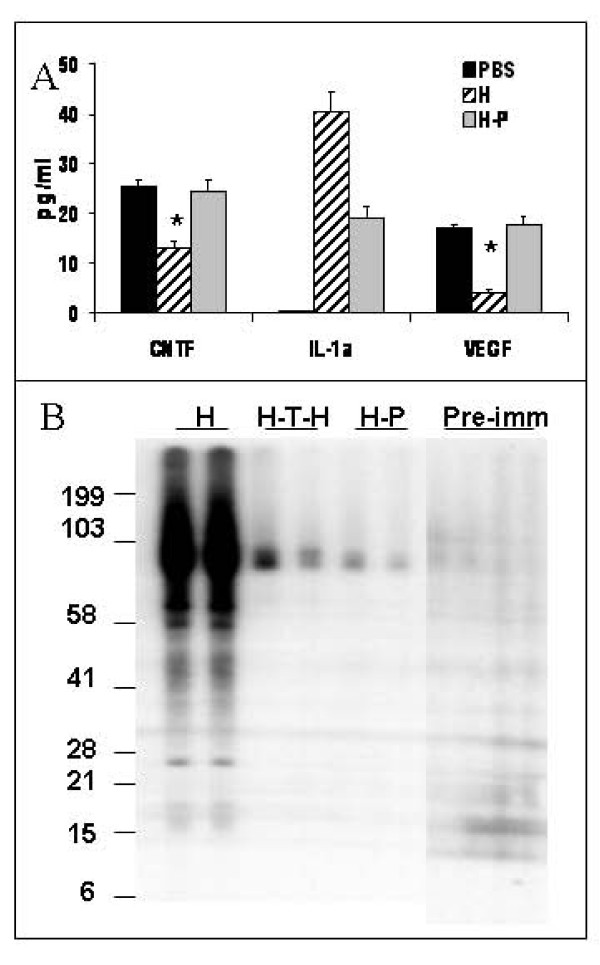
**Effects of Immunomodulatory Strategies on Rat Lung Cytokine levels and AECA production**. **(A) **Levels of IL-α, VEGF and CNTF in whole lung tissue homogenates from PBS-treated controls, HUVEC-immunized and pristane-pretreated/HUVEC-immunized rats measured by Quantibody Rat-1 Array. *p < 0.01 when compared to the untreated HUVEC-immunized rats. **(B) **Antibody production in HUVEC-immunized rats. HUVEC protein extracts were separated on 4-12% gradient Bis-Tris gel and WB was performed using serum (at 1:1000 dilution) from HUVEC-immunized (H) rats (lanes 1 and 2); intrathymically HUVEC-injected and HUVEC-immunized (H-T-H) rats (lanes 3 and 4); pristane pre-treated/HUVEC-immunized (H-P) animals (lanes 5 and 6); and pre-immune serum (lanes 7-10).

To examine whether induction of immune deviation by pristane can affect lung cytokine levels in lung tissue homogenates, we used Quantibody Rat-1 Array that allows measurements of the following cytokines: cytokine induced neutrophil chemoattractant-2 (CINC-2 also known as CXCL3/GROgamma/DCIP-1); cytokine induced neutrophil chemoattractant-3 (CINC-3 also known as CXCL3/GRObeta/MIP-2); Granulocyte-Macrophage Colony Stimulating Factor (GM-CSF); lipopolysaccharide-induced CXC chemokine (LIX also known as CXCL5); interleukin-4 (IL-4); interleukin 1alpha (IL-1α); ciliary neurotrophic factor (CNTF); Fractalkine (CX3CL1), nerve growth factor beta (β-NGF) and vascular endothelial growth factor (VEGF). HUVEC-immunization resulted in a greater than 40-fold increase in IL-1α, and a decrease in vascular endothelial growth factor (VEGF) and CNTF, levels (Figure 5A), whereas pretreatment with pristane preserved the normal VEGF and CNTF levels in the lung and significantly decreased IL-1α, levels in the HUVEC-immunized animals.

### Immunomodulation strategies protect abolished AECA production

Earlier we have demonstrated [14], that serum from HUVEC-immunized animals contains antibodies that bind to several HUVEC proteins. Induction of central tolerance achieved by intrathymic injection of HUVEC prior to IP immunization or immunomodulatory therapy with pristane abolished antibody production in the treated rats (Figure 5B). Pre-immune serum did not cross-react with HUVEC proteins. Thus, these findings suggest that the central and peripheral immunomodulatory strategies resulted in a reduction in lung inflammation characterized by a reduced recruitment of macrophages, CD4+ T cells and B cells to the rat lung in response to intraperitoneal HUVEC injection.

## Discussion

It has been shown by several groups that alveolar septal cells in the lungs from patients with severe emphysema are undergoing apoptosis [29,33,34], and that induction of apoptosis in mouse lungs indeed causes emphysematous changes [35]. Alveolar septal cell apoptosis is likely the consequence of oxidative stress [36], withdrawal of the impact of lung cell survival factors [28] and/or the action of proteases [37]. We have recently shown that alveolar septal cell apoptosis also occurs in our rat autoimmune emphysema model [14]. Here we apply established immune modulating strategies [19,38,39] to demonstrate that immunity-skewing approaches can modify the autoimmune response to HUVEC immunization, reduce lung cell apoptosis and prevent airspace enlargement.

The thymus plays an important role in acquired tolerance [40], and several studies show that intrathymic injection of alloantigens or autoantigens induces specific systemic tolerance in experimental autoimmune models [17,41]. The mechanism by which intrathymic injection of antigen results in systemic tolerance is still elusive, although injection of antigen into the thymus induces apoptosis of thymic T cells [42]. In addition, an increase in clonotype positive T cells in the thymus prevents the peripheral expansion of antigen-specific CD4+ T cells [43] and induces prolonged anergy [44].

The CD4^+ ^CD25^+ ^regulatory population of T cells (Treg cells), which expresses the forkhead family transcription factor (Foxp3), is the key component of the peripheral tolerance mechanism that protects against a variety of autoimmune diseases [45]. Here we demonstrate that intrathymic injection of HUVEC into 6 week old rats prior to the immunization results in the expansion of CD4+CD25+Foxp3+ Treg cells, suggesting that an expansion of this regulatory T cell subset may be one of the mechanisms responsible for the protective effect against emphysema development. Whether the mechanism of action for thymic inoculation of HUVEC is attributable to the intrathymic deletion of endothelial-reactive T cells or to the increased production of clonotype-positive T cells is the subject of ongoing investigations by our group.

In the present experiments, pristane modulated the immune response triggered by HUVEC immunization and prevented AECA production as well as lung cell apoptosis and emphysematous lung destruction. Pristane is considered as an immunological adjuvant namely in vaccines and while administration of the high doses of the hydrocarbon oil adjuvant pristane are used to induce lupus in a murine model of systemic lupus erythematosus [46] it also has been also shown to induce a persistent decrease in phytohemagglutinin responsiveness, consistent with a block of polyclonal T cell proliferation [18]. As an immune adjuvant, we expected pristane to produce immune skewing. We considered that Toll-like Receptor (TLR) signaling, which is activated by pristane (e.g. requirement of TLR 7 for pristane-induced production of autoantibodies and development of murine lupus nephritis [47]) would be upregulated and therefore immune injury could have been ramped up in the lungs. We did not know at the outset whether pristane would exacerbate autoimmunity or ameliorate it and were surprised about the experimental results.

Similar to human emphysema [48], T cells recruited to the lung in response to HUVEC might express a restricted TCR repertoire, suggesting their recruitment to lung in response to a conventional antigenic stimulus. We queried whether alterations of the TCR repertoire of lung and spleen CD4+ T cells had occurred as a result of pristane treatment. Our earlier studies did not find significantly expanded TCR-Vbeta subsets in ex vivo human COPD lung samples [48]. However, when cultured in vitro, we found seven major CD4-expressing TCR-Vbeta subset expansions from five of the patients with emphysema. This suggested that severe emphysema is associated with inflammation involving T lymphocytes that are composed of oligoclonal CD4+ T cells. Here we show that pristane treatment decreased the percentage of CD4+ T cells expressing TCR Vβ16.1 in the lung compared to the expression of Vβ16.1 on CD4+ T cells from the lungs of HUVEC-immunized rats. It has been reported that treatment with antibody directed against this receptor can facilitate prolonged graft survival [49], suggesting pathogenic nature of rat TCR Vβ16.1. We would like also to point out that while human and mouse TCR nomenclature is well characterized in the Immunogenetics Information System http://www.imgt.org, rat TCR are not in the system making it difficult to compare rat TCR repertoire with known nomenclatures and species.

As with VEGF receptor blockade-induced emphysema [28], HUVEC immunization also leads to a decrease in VEGF protein levels in the lung (Figure 5B). VEGF is likely a critical component of the lung structure maintenance program [50]. Here we demonstrate that pretreatment with pristane abolished antibody production and preserved normal levels of VEGF in HUVEC-immunized animals. Moreover, our data indicate that HUVEC immunization induced the expansion of CD4+ T cells expressing TCR Vβ16.1, which usually are associated with an allergic response [51]. This expansion was significantly blunted upon pre-treatment with pristane, suggesting that this T cell subset may be the pathogenic CD4+ T cell subset in this immune model of emphysema. We also found that the expression of ciliary neurotrophic factor (CNTF) was reduced in HUVEC-immunized rat lungs (Figure 5A). CNTF is a cytokine of the interleukin-6 (IL-6) family and its mRNA is widely expressed in the brain, heart, lung, and liver, of the rat, in addition to preferential expression in the sciatic nerve [52]. To our knowledge, this is the first report that CNTF protein expression is reduced in lungs of HUVEC immunized rats, and that immunomodulation with pristane prevents this decrease.

Interleukin-1α (IL-1α) is a 19 kDa proinflammatory cytokine that is a potent mediator of the body's response to inflammation, microbial invasion, tissue injury, and immunological response [53], and has a role in arthritis, Alzheimer's disease and tumor growth [54,55]. In this study, we demonstrated a significant upregulation of IL-1α levels in the HUVEC-immunized rat lung that was prevented by pristane treatment (Figure 5A).

Earlier [14] we have demonstrated that HUVEC injection resulted in the production of antibodies against endothelial cells. Here we show that in the lungs of HUVEC-injected rats, the number of B cells and the expression of cell surface CD19 is increased and that the immunomodulatory strategies attenuated this increase (Figure 3). Recently Sato and colleagues [56] reported that small changes in CD19 expression can induce autoantibody production and suggested that modest changes in the expression or function of regulatory molecules, such as CD19, may shift the balance between tolerance and immunity to autoimmunity [56]. It is possible that downregulation of CD19 in B cells, using immunomodulatory strategies applied by us, also reflects a shift toward tolerance in our model of autoimmune emphysema. However, it is also possible that the decrease in CD19 expression could simply reflect decreased numbers of B cells in the lungs each expressing normal levels of CD19. Taken together, the results of our experiments show that rats can be made tolerant to the effects of HUVEC-immunization.

A limitation of our studies is that we have not delineated which specific cell-cell interactions, cytokines, antioxidants or proteases are involved in developing tolerance and whether the different immunomodulatory strategies converge toward a final signaling pathway. However, we can conclude that - as there are multiple factors and conditions which can trigger lung emphysema development [12,57] - there are multiple interventions which can prevent experimental emphysema development.

Emphysema, until recently, has been seen as a problem of proteolysis, not of adaptive immunity. Whereas this is somewhat perplexing because the burning cigarette can be seen as an antigen delivery device, most recently a concept of emphysema pathogenesis based on immune mechanisms is emerging [11,12]. Lung gene expression profiling of cigarette smoke-exposed rats demonstrated a sustained increased expression of a number of genes implicated in the innate and adaptive immune responses [58]. Chronic lung cell damage, and in particular apoptosis when combined with ineffective phagocytosis (removal of apoptosed cell bodies) [59], may result in the generation of neoantigen peptides [60] and of autoantibodies. Moreover, tobacco anti-idiotypic antibodies have been identified in serum from smokers [61], and the recent finding of anti-elastin autoantibodies [13] in patients with tobacco smoking-induced emphysema, that correlated with severity of the disease, link emphysema to adaptive immunity against a specific lung antigen and suggest the potential for autoimmune pathology.

Whether "priming" strategies can also protect against other - non-HUVEC-induced forms of emphysema remains to be investigated. We are intrigued that experimental manipulations as diverse as intrathymic antigen injection and intraperitoneal delivery of pristane prevented the accumulation of lung B cells and macrophages, facilitated the expansion of CD4+CD25+Foxp3+ Treg cells and suppressed the expansion of TCR Vβ16.1 expressing T cells in the HUVEC-immunized animals. Whether or not TCR Vβ16.1 expressing T cells are associated with autoimmune emphysema still need to be verified. Whether or not this is true in human disease needs to be determined. Also, the role of macrophages in this autoimmune emphysema model deserves further investigation.

## Conclusions

The experimental autoimmune emphysema may evolve from a confluence of subtle multigenic variations in cell surface signaling molecules. As in NOD diabetes, where interventions prior to the development of diabetes skew immune responses and are associated with decreased inflammation around pancreatic islets and prevent diabetes [62,63], here we demonstrate that similar immunomodulatory strategies that suppress autoreactive T and B cells and upregulate Treg cells can prevent experimental autoimmune emphysema.

## List of Abbreviations

(AECA): Anti-endothelial cell antibodies; (HUVEC): human umbilical vein endothelial cells; (NOD): non-obese diabetic; (MLI): mean linear intercept; (TUNEL): Terminal deoxynucleotidyl transferase-mediated dUTP nick end-labeling; (TCR): T cell Receptor; (Treg): T regulatory cells; (IHC): immunohistochemistry; (VEGF): vascular endothelial growth factor; (CNTF): neurotrophic factor; (IL-1α): interleukin 1alpha; (CINC-2 also known as CXCL3/GROgamma/DCIP-1): cytokine induced neutrophil chemoattractant-2; (CINC-3 also known as CXCL3/GRObeta/MIP-2): cytokine induced neutrophil chemoattractant-3; (GM-CSF): granulocyte-Macrophage Colony Stimulating Factor; (IL-4): interleukin-4; (LIX/CXCL5): lipopolysaccharide-induced CXC chemokine; (β-NGF): nerve growth factor beta; (CX3CL1): Fractalkine.

## Competing interests

The authors declare that they have no competing interests.

## Authors' contributions

MH conducted the experiments, drafted the manuscript and performed statistical analysis; MRN participated in study design; APF participated in study design; DK participated in the animal experiments; DGM performed flow cytometry and data analysis; AK and JS have been involved in work leading to the revised the manuscript; VK participated in data acquisition; NB carried out western blots; NFV participated in the design of the study and helped to draft the manuscript and LT-S. conceived of the study and participated in its design and coordination and helped to draft the manuscript. All authors read and approved the final manuscript.
